# Phosphatidylcholine-fatty Alcohols Equilibria in Monolayers at the Air/Water Interface

**DOI:** 10.1007/s00232-015-9793-x

**Published:** 2015-03-24

**Authors:** Agnieszka Serafin, Zbigniew Artur Figaszewski, Aneta Dorota Petelska

**Affiliations:** 1 Institute of Chemistry, University of Bialystok, K. Ciolkowskiego Street 1K, 15-245 Bialystok, Poland; 2 Laboratory of Electrochemical Power Sources, Faculty of Chemistry, University of Warsaw, Pasteur St. 1, 02-093 Warsaw, Poland

**Keywords:** Phosphatidylcholine, Fatty alcohols, Complex formation equilibria, Monolayer, Langmuir trough

## Abstract

Monolayers of phosphatidylcholine (PC), tetradecanol (TD), hexadecanol (HD), octadecanol (OD) and eicosanol (E) and their binary mixtures were investigated at the air/water interface. The surface tension values of pure and mixed monolayers were used to calculate *π*–*A* isotherms. The surface tension measurements were carried out at 22 °C using a Teflon trough and a Nima 9000 tensiometer. The interactions between phosphatidylcholine and fatty alcohols (tetradecanol, hexadecanol, octadecanol, eicosanol) result in significant deviations from the additivity rule. An equilibrium theory to describe the behavior of monolayer components at the air/water interface was developed in order to obtain the stability constants, Gibbs free energy values and areas occupied by one molecules of PC–TD, PC–HD, PC–OD and PC–E complexes. We considered the equilibrium between the individual components and the complex and established that phosphatidylcholine and fatty alcohols formed highly stable 1:1 complexes.

## Introduction

Many studies have been conducted on the mixed monolayer behavior at the air/water interface. Investigating the surface properties of mixed monolayers is very important since it allows one to obtain information on the molecular interactions between the monolayer components. An insoluble monolayer at the air/water interface is usually considered as a two-dimensional solution, and the surface properties of mixed monolayers are generally studied based on the measurement of the surface pressure–area per molecule (*π*–*A*) isotherms of the monolayers (Capuzzi et al. 1997; Bordi et al. 1999; Chou and Chang 2000; Petelska et al. 2011, 2012, 2013).

Fatty alcohols are known to self-assemble at the air/water interface to form monolayers with rich phase behavior. While single-component alcohol monolayers have been well studied, mixtures have not. Mixtures are widely used as stabilizers for emulsions and foams, and are important as model biological membranes. The behavior of mixtures of PC with long chain fatty alcohols or amines is very similar to that of fatty acid–phospholipids mixture, with evidence for 1:1 complex formation (Boggs et al. 1986).

Mixed monolayer behavior of dipalmitoyl phosphatidylcholine (DPPC) with various long-chain alcohols at the air/water interface has been investigated by Kurtz et al. 2006, 2008 and Li et al. 2008. The results are of special interest for potential applications in the lung surfactant therapy field, because DPPC and C_16_OH constitute the major part of the artificial lung surfactant formulation, Exosurf (Clements 1982, 1989; Durand et al. 1985). With regard to the therapeutic use of DPPC:C_16_OH mixtures, it appears important to obtain more generalized information concerning the two-dimensional molecular interactions between DPPC and various long-chain alcohols as mixed monolayers at the air:water interface. A study to elucidate the effects of hydrocarbon chain length of long-chain alcohols on the miscibility and stability of mixed DPPC:long-chain alcohol monolayers may provide an insight on how to improve the surface properties of available surfactant therapy formulations.

Fatty alcohols are present in small amounts in biological membranes and at the same time also possess quite simple structure; they are frequently used by researchers for modeling. It is clear that there is certain equilibrium between lipids forming monolayer. The interactions between fatty alcohols and lipids are studied by several techniques (Huang et al. 1996; Chen et al. 2000, 2003; Kurtz et al. 2006, 2008; Li et al. 2008); however, there is still the lack of the quantitative description of the systems. Formation of artificial membranes with built-in studies components allows to research in simpler systems than complicated biological membranes.

The aim of the present work was to examine the possible effect of fatty alcohols (FA) component on the phosphatidylcholine monolayer and the molecular interaction between fatty alcohols (tetradecanol, hexadecanol, octadecanol, eicosanol) and phosphatidylcholine by analyzing physicochemical properties for binary mixed monolayers treated as the simplest model of a half of the biological membrane. In this paper we present evidence for the formation of 1:1 PC–FA complexes at the air/water interface and calculate their stability constants and Gibbs free energy values. The knowledge of stability constants and Gibbs free energy values of phosphatidylcholine-fatty alcohols systems let us understand the processes that take place both in monolayer itself and also on its surface.

Data presented in this work, obtained in result of mathematical derivation and confirmed experimentally, are of great importance for interpretation of phenomena occurring in lipid membranes. A quantitative description of equilibria between phosphatidylcholine and fatty acids lets us understand the processes that take place on membrane surface. The equilibria are particularly significant from the standpoint of cell functioning. Phosphatidylcholine-fatty alcohols interactions modulate a range of physicochemical properties of membranes and they are important in the course of the multiple processes involving membranes in the living cell (e.g. transport mechanism). Solution even the smallest problem of biological membranes, enriches knowledge of their properties and functioning, thereby moving us to a better understanding of many processes in a human organism.

## Theory

During formation of a mixed two-component monolayer on a free electrolyte surface, the individual components: phosphatidylcholine (denoted by PC) and fatty alcohols (denoted by FA) can form complex. The equilibrium of such a system is described by the complexation reaction. Two substances can form complexes of varying stoichiometry. However, due to the fact that the first stability constant in complexes is usually the largest (Inczedy 1976), we assumed that 1:1 complexes were predominant.

Let us assume that a 1:1 complex is formed in a mixed monolayer at the air/water interface between phosphatidylcholine and fatty alcohols (tetradecanol, hexadecanol, octadecanol and eicosanol). The reaction:

$${\text{PC}} + {\text{FA}} \Leftrightarrow {\text{PC}} - {\text{FA}}$$

may be described by the system of equations (Petelska and Figaszewski 2009, 2011, 2013):

1 $$a_{\text{PC}} S_{\text{PC}} + a_{\text{FA}} S_{\text{FA}} + a_{{{\text{PC}} - {\text{FA}}}} S_{{{\text{PC}} - {\text{FA}}}} = 1$$

2 $$a_{\text{PC}} + a_{{{\text{PC}} - {\text{FA}}}} = c_{\text{PC}}$$

3 $$a_{\text{FA}} + a_{{{\text{PC}} - {\text{FA}}}} = c_{\text{FA}}$$

4 $$K_{{{\text{PC}} - {\text{FA}}}} = \frac{{a_{{{\text{PC}} - {\text{FA}}}} }}{{a_{\text{PC}} \cdot a_{\text{FA}} }}$$

5 $$x_{\text{FA}} = \frac{{c_{\text{FA}} }}{{c_{\text{PC}} + c_{\text{FA}} }}$$

where $a_{\text{PC}}$, $a_{\text{FA}}$ and $a_{{{\text{PC}} - {\text{FA}}}}$ (mol m^−2^) are the surface concentrations of phosphatidylcholine, fatty alcohol and phosphatidylcholine-fatty alcohol complex; $c_{\text{PC}}$ and $c_{\text{FA}}$ (mol m^−2^) are the total surface concentrations of phosphatidylcholine and fatty alcohol; $S_{\text{PC}}$, $S_{\text{FA}}$ and $S_{{{\text{PC}} - {\text{FA}}}}$ (m^2^ mol^−1^) are the surface areas occupied by 1 mol of phosphatidylcholine, fatty alcohol and phosphatidylcholine-fatty alcohol complex; $K_{{{\text{PC}} - {\text{FA}}}}$ (m^2^ mol^−1^) is the stability constant of phosphatidylcholine-fatty alcohol complex, and $x_{\text{PC}}$ and $x_{\text{FA}}$ are the mole fractions of phosphatidylcholine and fatty alcohol.

The presented above system of Eqs. (1–5) contain unknown quantities $a_{\text{PC}}$,$a_{\text{FA}}$, $a_{{{\text{PC}} - {\text{FA}}}}$, $S_{{{\text{PC}} - {\text{FA}}}}$ and $K_{{{\text{PC}} - {\text{FA}}}}$ as well as known or easy to determined quantities $S_{\text{PC}}$, $S_{\text{FA}}$, $x_{\text{FA}}$, $c_{\text{PC}}$ and $c_{\text{FA}}$.

Attempts to solve this system of equations resulted in complicated expressions, so Eqs. (1–5) were differentiated with respect to x_FA_ and approximated to low or high argument values. At x_FA_ → 0 (a monolayer formed from pure phosphatidylcholine; x_PC_ → 1), and at x_FA_ → 1 (a monolayer formed from pure fatty alcohol; x_PC_ → 0), the system of Eqs. (1–5) are simplified, which was presented in earlier papers (Petelska and Figaszewski 2009, 2011, 2013).

Suitable transformations lead to expressions for two quantities of interest (each individually): the stability constant of the complex $K_{{{\text{PC}} - {\text{FA}}}}$ and the surface area occupied by one molecule of the complex $S_{{{\text{PC}} - {\text{FA}}}}$:

6 $$K_{{{\text{PC}} - {\text{FA}}}} = \frac{{S_{\text{FA}}^{3} c_{{{\text{FA}}(x_{\text{FA}} = 1)}}^{'} - 2S_{\text{PC}} S_{\text{FA}} - S_{\text{PC}}^{3} c_{{{\text{PC}}(x_{\text{FA}} = 0)}}^{'} }}{{S_{\text{FA}} - S_{\text{PC}} + S_{\text{PC}}^{2} c_{{{\text{PC}}(x_{\text{FA}} = 0)}}^{'} + S_{\text{FA}}^{2} c_{{{\text{FA}}(x_{\text{FA}} = 1)}}^{'} }}$$

7 $$S_{{{\text{PC}} - {\text{FA}}}} = \frac{{\left( {S_{\text{PC}} S_{\text{FA}} + c_{{{\text{PC}}(x_{\text{FA}} = 0)}}^{'} c_{{{\text{FA}}(x_{\text{FA}} = 1)}}^{'} S_{\text{PC}}^{2} S_{\text{FA}}^{2} } \right)\left( {S_{\text{PC}} + S_{\text{FA}} } \right)}}{{S_{\text{PC}}^{3} c_{{{\text{PC}}(x_{\text{FA}} = 0)}}^{'} + S_{\text{FA}}^{3} c_{{{\text{FA}}(x_{\text{FA}} = 1)}}^{'} }}$$

The slopes of tangent lines at the points x_FA_ → 0 and x_FA_ → 1 may be calculated from the following equations:

8 $$c_{{{\text{PC}}(x_{\text{FA}} = 0)}}^{'} = \frac{{K_{{{\text{PC}} - {\text{FA}}}} \left( {S_{\text{PC}} - S_{{{\text{PC}} - {\text{FA}}}} } \right) - S_{\text{PC}} S_{\text{FA}} }}{{S_{\text{PC}}^{2} \left( {S_{\text{PC}} + K_{{{\text{PC}} - {\text{FA}}}} } \right)}}$$

9 $$c_{{{\text{FA}}(x_{\text{FA}} = 1)}}^{'} = \frac{{ - K_{{{\text{PC}} - {\text{FA}}}} \left( {S_{\text{FA}} - S_{{{\text{PC}} - {\text{FA}}}} } \right) - S_{\text{PC}} S_{\text{FA}} }}{{S_{\text{FA}}^{2} \left( {K_{{{\text{PC}} - {\text{FA}}}} - S_{\text{FA}} } \right)}}$$

Equations (8) and (9) may be used for verification of slopes obtained either from theory or by experiment. Agreement between the slopes indicates that the method of calculating $K_{{{\text{PC}} - {\text{FA}}}}$ and $S_{{{\text{PC}} - {\text{FA}}}}$ is correct.

The phosphatidylcholine-fatty alcohol complex formation energy was calculated from Eq. (10):

10 $$- \log K = \frac{{\Delta G^{0} }}{2.3RT}$$

where $K$ (m^2^ mol^−1^) is the stability constant of phosphatidylcholine-fatty alcohol complex; $\Delta G^{0}$ (J mol^−1^) is the phosphatidylcholine-fatty alcohol complex formation energy; $R$ (J mol^−1^ K^−1^) is gas constant; $T$ (K) is the temperature.

## Materials and Methods

### Materials

Phosphatidylcholine from soybean (≥97 %, Roth) prepared by a modification of the procedure of Singleton et al. (1965) was used in experiment. Tetradecanol ≥95 %, hexadecanol ≥99 %, octadecanol ≥99 %, eicosanol ≥97 % were purchased from Fluka and were used as received. The molecular weights of the PC, TD, HD, OD and E were approximately 752.08, 214.39, 242.45, 270.50 and 298.56 g mol^−1^, respectively.

The 1-chloropropane solvent (>98 % pure) was supplied by Aldrich. Solutions were prepared by dissolving appropriate amounts of each material in 1-chloropropane at a concentration of 1 mg cm^−3^ and were stored at 4 °C. The water used in the experiments was prepared by triple distillation (the second distillation was performed over KMnO_4_ and KOH to remove organic impurities).

### Methods

The homemade computer-controlled apparatus used for surface tension measurements was presented in previous paper (Petelska and Figaszewski 2009).

The surface tension measurements were carried out at the water/air interface at 22 °C, and were expressed as surface pressure-area per molecule (*π*–*A*) isotherms. For all experiments, the trough was filled with triple-distilled water as the subphase. The monolayers were prepared by spreading a defined volume of a lipid solution in 1-chloropropane on the aqueous subphase using a Hamilton micro-syringe. Ten minutes were allowed for solvent evaporation and monolayer equilibration before an experiment was begun. The monolayer was continuously compressed to obtain the *π*–*A* isotherms using the glass barrier. The Nima ST9002 computer program was used to calculate the surface pressure of the monolayer *π* as a function of surface area per molecule A: *π* = *γ* − *γ*_*0*_ = *f*(*A*), where *γ* is the surface tension of the lipid-covered surface and *γ*_*0*_ is the surface tension of the bare air/water interface.

Before each trial the Teflon trough (trough size 648 cm^2^) was washed and rinsed with purified water. The subphase surface was cleaned just prior to each measurement by suction with a vacuum pump until the surface tension was constant and equal to the surface tension value of pure water at 22 °C (approximately 72 mN m^−1^). All glassware in contact with the samples was cleaned with chromic acid and repeatedly rinsed with purified water before use.

The system was enclosed in an acrylic box to minimize water evaporation, to ensure high humidity, and to avoid contamination of the system.

All of the reported values are highly reproducible and represent the average of at least five experiments. The standard deviation for surface area measurements was less than 1 %.

## Results and Discussion

In this paper we present evidence for the formation of 1:1 phosphatidylcholine-fatty alcohol complexes at the air/water interface. Using equations from “Theory” section, the stability constants and Gibbs free energy of the PC–TD, PC–HD, PC–OD and PC–E complexes were calculated. This paper contains the first report of stability constants and Gibbs free energies for PC–TD, PC–HD, PC–OD and PC–E complexes in monolayer.

**Fig. 1:**
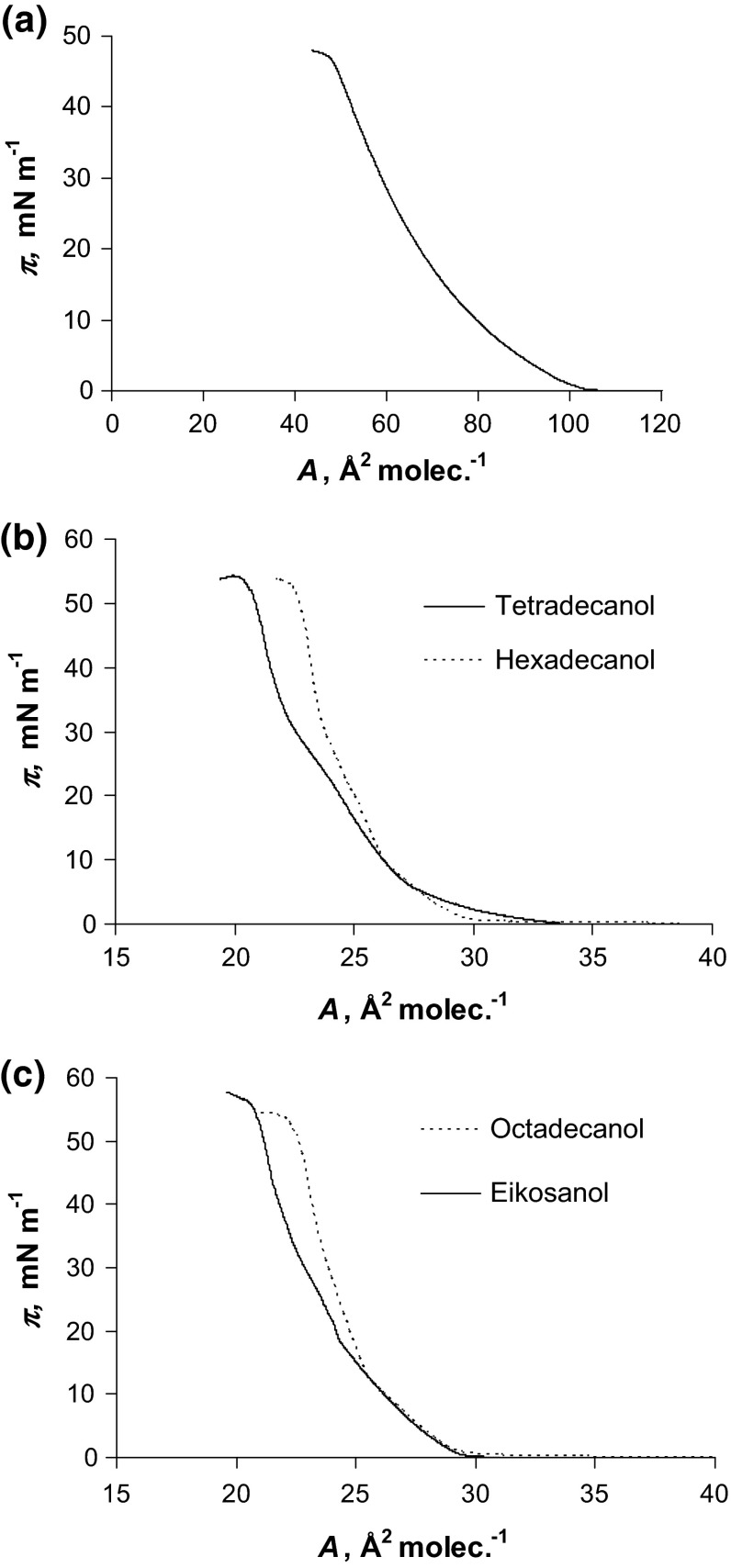
Isotherms of phospatidylcholine (**a**), tetradecanol and hexadecanol (**b**) and octadecanol and eicosanol (**c**)

Figure 1a presents *π*–*A* isotherms of phosphatidylcholine (1), tetradecanol and hexadecanol (b), octadecanol and eicosanol (c). The phosphatidylcholine isotherm (Fig. 1a) is in satisfactory agreement with that previous reported (Brzozowska and Figaszewski 2002; Sovago et al. 2007). With decreasing surface area, the following regions have been indicated: a gas phase, the liquid condensed phase and the pure condensed phase (Brzozowska and Figaszewski 2002; Gzyl and Paluch 2004; Sovago et al. 2007). Lecithin monolayer is an example of liquid-expanded membrane, with the hydrophilic head groups located in the aqueous subphase and the hydrophobic fatty acid tails oriented toward the air. The surface area per lipid molecule assumes various values depending on the length, conformation, and degree of unsaturation of the hydrocarbon chains. The surface area for the lecithin molecule in pure water is 78 Å^2^. This value is agreed with the reported in literature 45–96 Å^2^ (Joos and Demel 1969; Jain 1972; Smondyrev and Berkowitz 1998; Tien and Ottova-Leitmannova al. 2003; Gzyl and Paluch 2001, 2004; Sovago et al. 2007; Petelska et al. 2008; Petelska and Figaszewski 2009).

The slopes of fatty alcohols (Fig. 1b, c) isotherms are very high, indicating a perpendicular orientation of the molecules at the interface with the hydrophilic group directed at the aqueous subphase. The fatty alcohol isotherms are in satisfactory agreement with previous reported (Chen et al. 2000; Kurtz et al. 2006, 2008; Li et al. 2008). The surface areas for tetradecanol, hexadecanol, octadecanol and eicosanol molecules ~24–26 Å^2^ molecule^−1^ (Fig. 1) were obtained experimentally by extrapolating isotherms to *π* = 0. This is in agreement with the previously reported values (Chen et al. 2000; Kurtz et al. 2006, 2008; Li et al. 2008).

### Phosphatidylcholine-fatty Alcohol Complexes

An equilibrium theory, presented in “Theory” section, to describe the behavior of monolayer components at the air/water interface was developed in order to obtain the stability constants and Gibbs free energy values of PC–TD, PC–HD, PC–OD and PC–E complexes. Table 1 lists several physicochemical parameters for monolayers containing PC–TD, PC–HD, PC–OD and PC–E complexes.

**Table 1:** Selected physicochemical parameters for four complexes: phosphatidylcholine-tetradecanol (PC–TD), phosphatidylcholine-hexadecanol (PC–HD), phosphatidylcholine-octadecanol (PC–OD), phosphatidylcholine-eicosanol (PC–E)

Examined system	Surface area occupied by one molecule of complex (Ǻ^2^ molecule^−1^)	Stability constant of examined complex (m^2^ mol^−1^)	Complex formation energy (Gibbs free energy) (kJ mol^−1^)
PC–TD	101 ± 1	1.08 × 10^6^	−34 ± 2
PC–OD	100 ± 1	1.10 × 10^6^	−34 ± 2
PC–HD	100 ± 1	1.13 × 10^6^	−34 ± 2
PC–E	99 ± 1	1.15 × 10^6^	−35 ± 2

The area per PC–TD, PC–HD, PC–OD and PC–E complexes and their stability constants were calculated by inserting the experimental data into Eqs. (6, 7). It should be emphasized that the stability constants values of phosphatidylcholine-fatty alcohols complexes presented in Table 1 are similar to the stability constants values of phosphatidylcholine-fatty acids values (Petelska and Figaszewski 2011) and are equal around 10^6^ m^2^ mol^−1^.

The surface areas occupied by one molecule of complexes value obtained this way are higher than the areas of PC molecule, *S*_PC_ = (78 Å^2^ molecule^−1^), but slightly lower than the sum of the areas of phosphatidylcholine and fatty alcohols (for example: *S*_PC_ + *S*_TD_ = 101 ± 1 Å^2^).

Using the values calculated for *S*_PC–FA_ and *K*_PC–FA_ in Eqs. (8, 9), theoretical c′_PC_ and c′_FA_ values were calculated and compared with the slopes of lines tangent to the experimental data at points x_FA_ → 0 and x_FA_ → 1.

Analysis of the results presented in Table 1 leads to the following conclusions:

1. The stability constant of the PC–TD complex is 1.08 × 10^6^ m^2^ mol^−1^, whereas the stability constant of the PC–HD, PC–OD and PC–E complexes are 1.10 × 10^6^, 1.13 × 10^6^ and 1.15 × 10^6^ m^2^ mol^−1^, respectively. These values are relatively high, providing additional support for the prevalence of 1:1 complexes in mixed monolayers.

2. The experimentally obtained values for the areas occupied by the PC–TD, PC–HD, PC–OD and PC–E complexes are almost identical and are equal ~99–101 ± 1 Å^2^ molecule^−1^.

3. The complex formation energy (Gibbs free energy) values for the PC–TD PC–HD, PC–OD and PC–E complexes are ~34–35 ± 2 kJ mol^−1^.

4. Because the examined compounds have similar cross-sectional area, the surface areas occupied by one molecule in monolayer are also similar (Fig. 1). Irving Langmuir first found that the surface area of the saturated fatty acid corresponds to the cross section area of the hydrocarbon chain, and does not depend on the length (Langmuir 1917). In the case of examined fatty alcohols we noticed the same relationship as for of fatty acids (Fig. 1; Table 1).

**Fig. 2:**
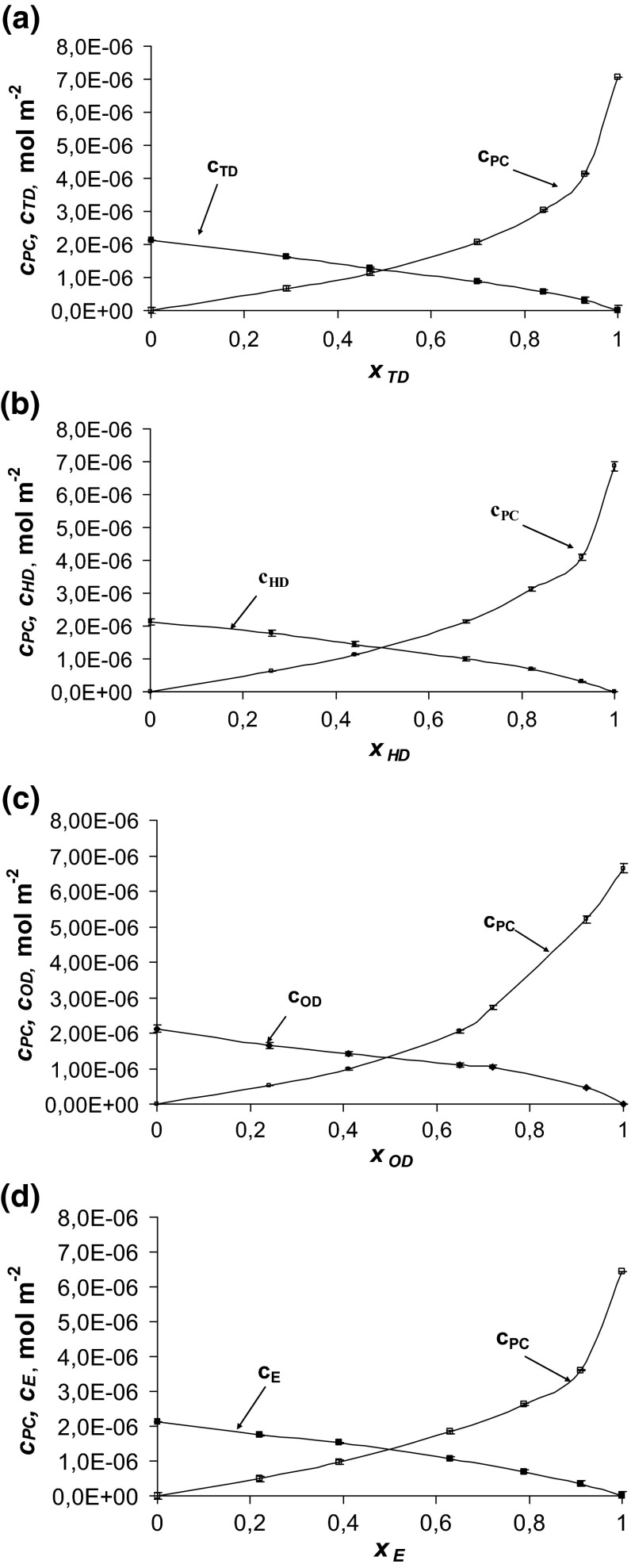
The dependence of total surface concentration of phosphatidylcholine (c_PC_) and fatty alcohol (c_FA_) on the mole fraction of fatty alcohol: (**a**) PC-tetradecanol, (**b**) PC-hexadecanol, (**c**) PC-octadecanol and (**d**) PC–Eicosanol systems (the experimental values are indicated by *points* and the theoretical values by the *curves*)

The total surface concentrations of phosphatidylcholine and fatty alcohols versus mole fraction of fatty alcohols are depicted in Fig. 2a–d. The nearly linear shape of the *c*_FA_ = *f* (*x*_FA_) function confirms the condensed character of the membrane (Birdi 1989). The condensation effect of fatty alcohols describes the decrease in surface area per phospholipid molecule in the monolayer in the presence of fatty alcohols (Chen et al. 2000; Li et al. 2008).

The presence of tetradecanol, hexadecanol, octadecanol and eicosanol with a comparatively small head group in the two-tailed PC monolayer at the interface could result in better molecular packing and attractive interaction between the molecules, showing a condensing effect similar to that observed in mixed phosphatidylcholine-cholesterol monolayers (Brzozowska and Figaszewski 2002).

Chen et al. (2000) from a detailed analysis of surface pressure–area isotherms are conclude that DPPC and normal long-chain alcohols with chain length of 16, 18 and 20 were miscible and formed non-ideal monolayers at the air/water interface. The Authors postulate that the hydrophobic interactions were dominant in the molecular packing of the mixed monolayers at the lower surface pressure. However, the packing efficiency or geometric accommodation may favor the molecular packing of mixed DPPC/C_16_OH monolayers at higher surface pressure, resulting in unexpected stronger effects of C_16_OH than C_18_OH on a DPPC monolayer.

The behavior of mixed monolayers also can be analyzed by comparing their *π*–*A* isotherm with the corresponding theoretical isotherm in which no interactions between the molecules are assumed by the additivity rule (Gzyl and Paluch 2004):

11 $$A_{12} = x_{1} A_{1} + x_{2} A_{2}$$

where: $A_{ 1 2}$ corresponds to the mean molecular area of the mixture at the fixed surface pressure, $A_{ 1}$ and $A_{ 2}$ are the molecular areas of pure components 1 and 2 at the same given surface pressure, and $x_{ 1}$ and $x_{ 2}$ are the molar fractions of components 1 and 2, respectively.

From Fig. 2 one can easily see the deviation from ideal behaviors which are indications of two-dimensional miscibility of the two components at the air/water interface. The deviations may be due to the geometrical rearrangement of the two components, which results in changes in area, thus causing enhanced dispersive interactions.

**Fig. 3:**
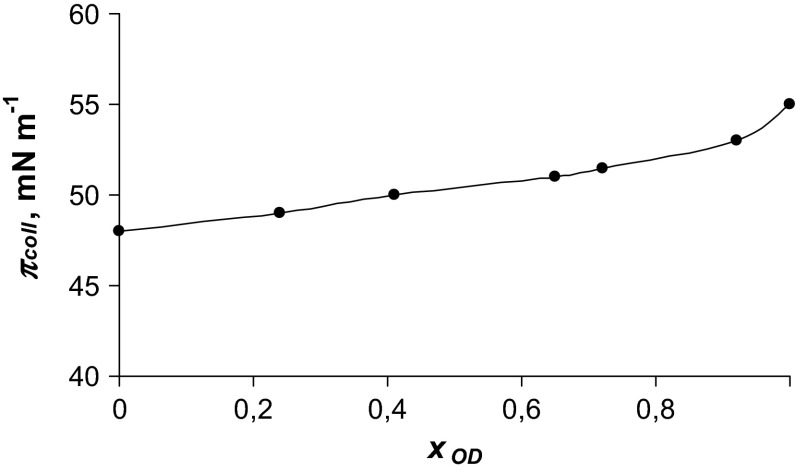
Collapse pressures versus molar fractions of octadecanol

Figure 3 presents collapse pressures versus molar fractions of octadecanol. For the other three studied systems (PC–TD, PC–HD and PC–E) the dependence shown in the Fig. 3 were also obtained. Due to the fact that the courses of these curves are similar, we presented the graph only for phosphatidylcholine-octadecanol system (Fig. 3).

The miscibility of films can also be deduced from the collapse characteristic. According to the phase rule first applied to the two-dimensional state by Crisp (1949), two miscible components have collapse pressures dependent on the mixture composition. Our results (Fig. 3) show that the collapse pressures of the mixtures change with composition. Thus, we conclude that phosphatidylcholine and fatty alcohols give true mixed films.

In Fig. 2a–d, the experimental points are compared with the values calculated using Eqs. 1–3 (depicted as lines). The theoretical values obtained are presented in Fig. 2a–d are marked by lines; points on the same figure show the experimental values. It can be seen that the agreement between experimental and theoretical points is very good, which verifies the assumption of the formation of the 1:1 complexes in the mixed PC–TD, PC–HD, PC–OD and PC–E monolayers. The lack of variation between theoretical and experimental point indicates that theoretical model (presented under “Theory” section, above) is sufficient to describe the interaction in phosphatidylcholine-fatty alcohol systems. The agreement between the experimental results and the model predictions for these systems justifies the statement that other complexes do not represent a significant component of these systems.

## Conclusion

This work continues systematic study of the lipid–lipid and lipid-other substance (for example: fatty acids, amines, amino acids) interactions realized by Petelska et al. (Petelska and Figaszewski 2009, 2011, 2013; Petelska et al. 2011). In this paper we present evidence for the formation of PC–FA complexes at the air/water interface and calculate their surface areas, stability constants and formation energy. These studies will ultimately aid in developing model for lipids, monolayer and bilayer formation, or other biological functions. In conclusion we would like to emphasize that the stability constants for phosphatidylcholine-fatty alcohol complexes in monolayers have been reported here for the first time.

We have shown that, as in the case of fatty acids the monolayer of fatty alcohols with different hydrocarbon chain lengths, demonstrate almost the same area per a single molecule. The same relationship is also noted in the analysis of the complexes formed between lecithin and fatty alcohols. Obtained values characterizing the examined complexes: stability constants, formation energies and areas occupied by a single molecule of lecithin-fatty alcohol complexes are similar (Table 1).
